# Dissolution and Absorption of Inhaled Drug Particles in the Lungs

**DOI:** 10.3390/pharmaceutics14122667

**Published:** 2022-11-30

**Authors:** Basanth Babu Eedara, Rakesh Bastola, Shyamal C. Das

**Affiliations:** 1 School of Pharmacy, University of Otago, Dunedin 9054, New Zealand; 2 Center for Translational Science, Florida International University, Port St. Lucie, FL 34987, USA

**Keywords:** dissolution, absorption, inhalation, dry powders, fine particle dose

## Abstract

Dry powder inhalation therapy has been effective in treating localized lung diseases such asthma, chronic obstructive pulmonary diseases (COPD), cystic fibrosis and lung infections. In vitro characterization of dry powder formulations includes the determination of physicochemical nature and aerosol performance of powder particles. The relationship between particle properties (size, shape, surface morphology, porosity, solid state nature, and surface hydrophobicity) and aerosol performance of an inhalable dry powder formulation has been well established. However, unlike oral formulations, there is no standard dissolution method for evaluating the dissolution behavior of the inhalable dry powder particles in the lungs. This review focuses on various dissolution systems and absorption models, which have been developed to evaluate dry powder formulations. It covers a summary of airway epithelium, hurdles to developing an in vitro dissolution method for the inhaled dry powder particles, fine particle dose collection methods, various in vitro dissolution testing methods developed for dry powder particles, and models commonly used to study absorption of inhaled drug.

## 1. Introduction

Although pulmonary drug delivery by inhalation has been used for many years, research in dry powder inhalers (DPIs) has undergone rapid advancements during the last decade for both local and systemic delivery of drugs [1,2,3,4]. DPIs are monophasic solid particulate mixtures, introduced in the 1970s. DPIs are easy to process, portable, more stable, eco-friendly due to the absence of propellants, patient compliance and cost-effective [5,6,7,8,9,10]. Most of the DPIs available in the market are suffering from short residence time and low drug bioavailability locally in the lungs, resulting in suboptimal local therapeutic effect [11,12].

The rapid dissolution of micron-sized particles and subsequent absorption of the drug into the systemic circulation is one of the clearance mechanisms of inhaled drug particles from the lungs [13,14,15]. Therefore, many formulation strategies have been followed to prolong the residence time of inhaled drugs at the site of action with reduced dosing and to avoid unwanted toxicities [16,17]. Some of the approaches to prolong the residence time of the inhaled drug particles in the lung are drug encapsulation in a particulate carrier system (liposomes, polymeric and lipid microparticles), increase the molecular mass of the drug by conjugating with a ligand and decrease the solubility of the drug by conjugation with a low water-soluble, hydrophobic material [18].

In vitro dissolution testing is a traditional and standardized quality control tool in all the pharmacopoeias used to evaluate the batch-to-batch consistency, differentiate immediate and controlled release formulations and also to approximate in vivo release profiles [19]. There are many well-established pharmacopeial dissolution methods for oral solid dosage forms, however, there is no accepted standardized method for inhaled products, although many dissolution methods for testing aerosols have been developed [20,21,22,23,24,25,26,27,28].

This review describes the dissolution of inhaled respirable size particles and absorption of dissolved drug through lung epithelium. It covers a summary of airway epithelium, hurdles to develop an in vitro dissolution method for the inhaled dry powder particles, fine particle dose collection methods, various in vitro dissolution testing methods developed for dry powder particles, and various models commonly used to study the absorption of inhaled drugs.

## 2. Airway Epithelium

**Figure 1:**
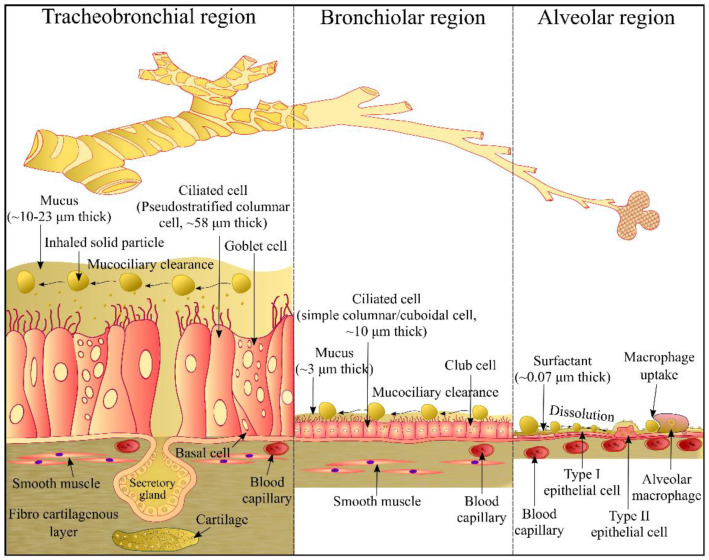
Comparison of the tracheobronchial, bronchiolar and alveolar regions of the lungs [32]. Reproduced with permission from Ref. [32]. 2015, McGraw Hill.

Dense core-granulated cells, basal cells, Clara cells, serous cells, ciliated cells, and mucus goblet cells are six distinct cell types present in the epithelium of the respiratory tract (Figure 1). At all levels of the airway, ciliated cells are the most abundant cells. Their primary function is to propel mucus towards the proximal direction, in simple term the process is known as mucociliary clearance. The ciliated cells in the bronchial pseudostratified epithelium are interspersed by secretory cells (mainly mucus-secreting goblet cells), whereas ciliated cells are interspersed mainly by Clara cells in the bronchiolar cuboidal epithelium. Two types of pneumocytes namely, type I and type II pneumocyte alveolar cells are found in the alveolar squamous epithelium (Figure 1). The luminal surface of the alveoli is mainly lined with alveolar type I cells. In addition, alveoli contain alveolar type II pneumocytes that possess microvilli and are cuboidal secretory cells [29]. Epithelial cells in the airway contribute to the secretion of respiratory tract lining fluid (RTLF) that lies on the surfaces of airways from nasal airways to alveolar regions [30]. RTLF is mainly composed of mucins in the conducting airways (trachea, bronchi, bronchioles and terminal bronchioles) whereas it mainly contains phospholipid-rich surfactants in respiratory zone (respiratory bronchioles, alveolar ducts and alveolar sacs) [31].

Particles inhaled in the respiratory tract have to overcome the non-epithelial pulmonary barriers (such as RTLF, mucociliary clearance, macrophage uptake) before they come in contact with the epithelial cells. Different types of transport systems occur in the epithelium of the airways such as paracellular transport, receptor-mediated transport and transporter-mediated transport [33]. Such transport systems translocate inhaled particles into epithelial cells and/or across the epithelia into the interstitium and to the blood and lymph [34].

## 3. In Vitro Dissolution Testing of Inhalable Dry Powder Particles

In vitro characterization of dry powder formulations includes the determination of physicochemical nature and aerosol performance of powder particles. The relationship between particle properties (size, shape, surface morphology, porosity, solid state nature, and surface hydrophobicity) and aerosol performance of an inhalable dry powder formulation has been well established [18,35,36,37,38]. However, unlike oral formulations, there is no standard dissolution method for evaluating the dissolution behaviour of the inhalable dry powder particles in the lungs.

### 3.1. Hurdles to Develop an In Vitro Dissolution Method for Inhalable Dry Powder Particles

One region of the lung differs from another in its anatomy and physiology (Figure 1). In addition, the RTLF where the inhaled particles dissolve varies regionally in composition, thickness and volume. A mucus gel (~3–23 µm) covers the airway region (trachea, bronchi, bronchioles) of the lungs over an area of 1–2 m^2^. Composition of the mucus gel includes 95% of water, 2–3% of mucins, 0.3–0.5% lipids, 0.1–0.5% non-mucin proteins and other cellular debris [39,40]. However, an extremely thin (estimated thickness ~0.07 µm) film of the lung surfactant covers the alveolar region (>100 m^2^) of the lungs. Lung surfactant contains 90.0% lipids (85.0% phospholipids: dipalmitoyl phosphatidylcholine (47.0%), unsaturated phosphatidylcholine (29.3%) and other lipids (23.7%); 5.0% neutral lipids: cholesterol) and 10.0% proteins (surfactant protein-SP) [41,42,43]. Hydrophilic SP comprises 3–5% SP-A, and <0.5% SP-D whereas hydrophobic SP contains 0.5–1.0% of SP-B and SP-C each. Gradual decrease in the thickness and volume of the RTLF along a respiratory tract is a major challenge for the development of an in vitro dissolution method that can accurately mimic the conditions of the lungs.

### 3.2. Fine Particle Dose (FPD) Collection

**Figure 2:**
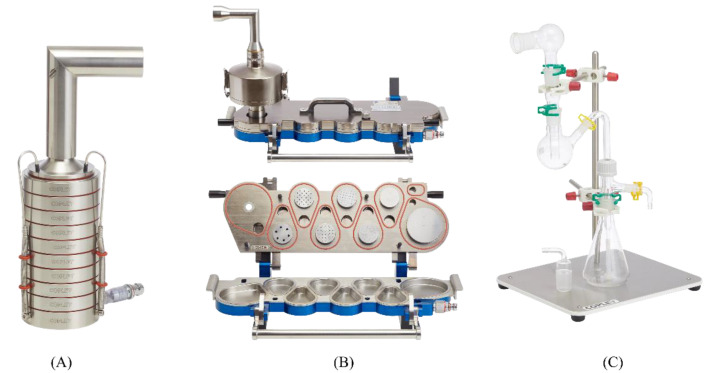
Various approaches to collect fine particle dose (FPD). (**A**) Andersen Cascade Impactor (ACI), (**B**) Next Generation Impactor (NGI; top- closed view and bottom- open view of NGI), and (**C**) Twin Stage Impinger (TSI). Figures (**A**–**C**) were reproduced with permission from Driving Results in Inhaler Testing [Brochure, 2020 edition] [46], Copley Scientific Limited.

During inhalation, only a fine particle dose (FPD) with the particle size 1–5 μm deposits in the deeper lung regions [44,45]. Therefore, estimation of the FPD dissolution profile seems to be more applicable than the whole dose of the powder formulation. To this end, the Andersen Cascade Impactor (ACI), Next Generation Impactor (NGI), Twin Stage Impinger (TSI) and PreciseInhale system (Figure 2) have been used to collect the fine particle dose (FPD). Table 1 summarizes the FPD collection methods for dissolution testing of respirable particles.

#### 3.2.1. Andersen Cascade Impactor (ACI)

The Andersen cascade impactor (ACI, Figure 2A) is one of the high flow rate cascade impactors used to assess the aerodynamic size distribution of particles for both pharmaceutical and toxicological applications [60]. It consists of a standard tubular induction port (IP) with a 90° curvature, stages from 0–7 and a filter stage (stage-F). Each impactor stage comprises several nozzles with a decreasing size as the stage number increases, which directs air and particles onto the collection plates.

Davies and Feddah, 2003 [21] collected dry powder particles onto a glass fibre filter at the connection point of the induction port and inlet part of ACI using a custom designed stainless steel ring with a stainless steel screen support filter. The ACI assembly consists of an induction port and base of the impactor with only stage number zero. The main drawback of this collection method is that the whole emitted dose is collected over the filter, which does not mimic the size of the particles deposited in the deeper lung regions.

Later, Arora et al., 2010 [20] collected aerodynamically classified particles with diameters of 4.7–5.8 μm and 2.1–3.3 μm on the filter membranes from stage 2 and stage 4 of ACI. In this study, they used a 8-stage ACI with stage 2 and stage 4 collecting plates turned upside down to arrange six polyvinylidene difluoride (PVDF) filter membranes (25 mm in diameter; 0.22 μm pore size) for dose collection.

In another study, May et al., 2012 [25] collected particles onto the regenerated cellulose membrane filters at stage-F of an abbreviated ACI. In this study, the ACI assembly consisted of an induction port, pre-separator, stages 0, 1 and F. Later they modified this assembly by adding a cylindrical stage extension of 5.8 cm in between stage 1 and stage-F to attain a homogenous particle distribution on the membrane [47]. Further, a modified filter stage comprising of only three small bars was used to change the flow and deposition pattern of aerosolized particles. The modifications in ACI resulted in homogenous deposition of particles on the membrane compared to unmodified ACI.

Rohrschneider et al., 2015 [26], collected aerosolized particles onto the filter papers positioned at stage 4 of an 8-stage ACI connected to an external humidifier to maintain the humidity.

#### 3.2.2. Next Generation Impactor (NGI)

Next generation impactor (NGI, Figure 2B) is a high flow rate cascade impactor specially designed for pharmaceutical inhaler testing. NGI was constructed with seven distinct stages plus a micro-orifice collector (MOC, a final filter) with a minimum stage overlap. The airflow passes with increasing velocity in a saw tooth pattern through a series of nozzles containing progressively reducing jet diameters. Out of seven, five stages give a particle size cut-off diameter of 0.54–6.13 μm at flow rates from 30 to 100 L min^−1^.

Son et al., 2009 [28], collected aerodynamically separated particles on a polycarbonate membrane (PC) using a modified NGI. At each collection plate of NGI, a polycarbonate (PC) membrane was placed and covered with a plate-shaped wax paper which consists of a rectangular opening (2.0 × 2.5 cm) at the centre. The powder samples were dispersed into the NGI using an Aerolizer^®^ device at an air flow rate of 60 L min^−1^ for 15 s per capsule. The limited size of the prototype holder frame only collects a fraction of powder particles over a rectangular area of the membrane. To overcome this, they designed a special membrane holder which fit in with the NGI cup and collected the whole dispersed particles [51,61]. However, the particles collected using the NGI either with a prototype holder frame or a special membrane holder were not homogenously distributed over the membrane.

#### 3.2.3. Twin Stage Impinger (TSI)

The Twin stage impinger (TSI, Figure 2C) is a simplified device to multistage liquid impinger with only two stages. It was developed to assess drug delivery from meter dose inhalers. TSI is made up of a series of glassware components such as an inlet, a glass bulb which simulates oropharynx, upper (stage 1) and lower (stage 2) impinger stages. TSI separates the actuated aerosol into a coarse oropharyngeal fraction (non-respirable fraction) and a fine pulmonary fraction with an aerodynamic diameter of ≤6.4 µm [60].

Grainger et al., 2009 [55] modified the TSI (mTSI) to deposit the respirable particles of dextrans onto the Calu-3 bronchial epithelial cells in a Transwell^®^ insert. A Transwell^®^ insert containing the cells was attached in the place of an adapter piece to the TSI conducting tube in the lower stage without any medium. The powder (~5 mg) was loaded into the dry powder insufflator and aerosolised at 60 L min^−1^ air flow rate for 5 s. The particles collected using the TSI are homogenously distributed with a geometric diameter of <6.4 µm. Later they used the same mTSI to collect the beclomethasone dipropionate (BDP) respirable particles for in vitro dissolution testing [56]. The BDP particles emitted from each of the commercial pressurized metered dose inhalers [pMDI): QVAR and Sanasthmax, were collected (1.2 ± 0.12 mg) on a hydrated nitrocellulose membrane (0.45 μm pore size).

Haghi et al., 2012 [57] collected the micronized salbutamol base (SB) and salbutamol sulfate (SS) particles using the mTSI as described by Grainger et al., 2009 [55] for in vitro dissolution studies using the Franz cell. Five milligrams of powder sample was actuated using a Cyclohaler DPI device at 60 L min^−1^ for 4 s. The emitted particles were collected in a Transwell^®^ polyester insert (0.4 μm pore size and 0.33 cm^2^ area) at stage-2 of mTSI.

**Figure 3:**
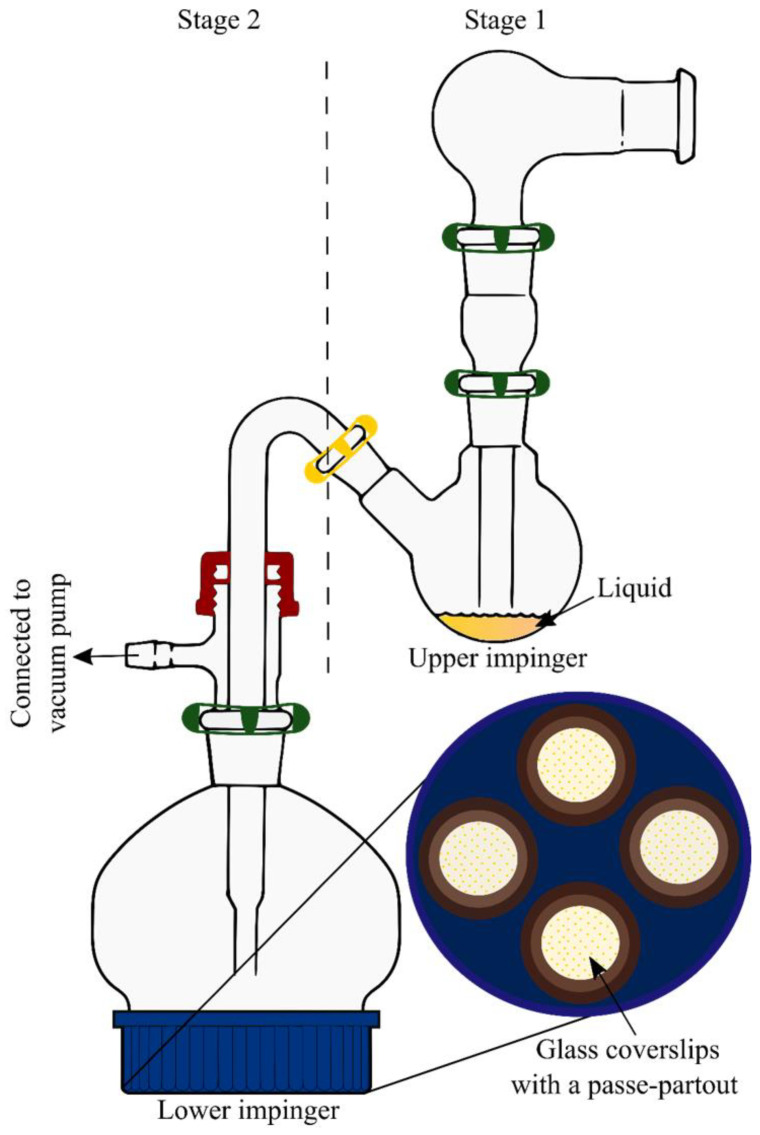
A modified Twin Stage Impinger (mTSI) to collect fine particle dose (FPD). Reproduced with permission from Eedara et al., 2019 [62], Springer Nature.

Eedara et al., 2019 [62] modified the stage 2 (lower impingement chamber) of TSI (mTSI, Figure 3) with a screw cap at its bottom to collect aerosolized powder particles onto the glass coverslips. Magnetic passe-partouts were used to hold glass coverslip (24 mm diameter) in position as it makes a boundary to collect the particles over an area of ~200 mm^2^ (16 mm diameter) during aerosolization. Hard gelatin PEG capsule (size 3; Qualicaps, Osaka, Japan) was used to fill the drug powders (20 mg). Capsule was dispersed using an Aerolizer^®^ device (Novartis, Surrey, UK) at a flow rate of 60 L min^−1^ for 4 s into stage 1 (upper impingement chamber) filled with 7 mL of water. The non-respirable fraction of dose gets separated in the stage 1 of TSI. Three capsules were actuated one after another and the mTSI was disassembled to collect coverslips with deposited powders.

In a recent study, a modified version of Twin Stage Impinger and in vitro dissolution experiment were used to examine in vitro in vivo correlation of budesonide and salbutamol. Comparison using both the actual and predicted in vivo pharmacokinetic values of the mentioned drugs and the pattern of their Concentration-Time profiles illustrated a good similarity [63].

#### 3.2.4. PreciseInhale System

The PreciseInhale system (Inhalation Sciences, Sweden) is a new aerosol delivery technique that is able to generate a dry powder aerosol in a free flowing state [64]. This system is a combination of a highly efficient aerosol generator and a precision dosing aerosol exposure unit. In brief, the powder to be aerosolized is placed in a powder chamber and suspended in a compressed gas passing from a pressure chamber to the powder chamber. The suspended powder agglomerates in the powder chamber ejects through the narrow conduit into a holding chamber with an ambient pressure and produces an aerosol cloud of deagglomerated particles. The aerosol of deagglomerated particles is transferred by airflow to the animal or collected for analysis.

Gerde et al., 2017 [23] collected the aerosolized dry powder particles of budesonide (BD) and fluticasone propionate (FP) on circular microscope glass coverslips using the PreciseInhale aerosol generator for in vitro dissolution testing by the Dissolv*It* system. Nine circular glass coverslips (13 mm diameter) were placed in a ring-shaped holder and covered with a thin steel passe-partout to limit the area of powder coating to the surface that will be in contact with the model barrier during the dissolution study. The coverslips were exposed to a single generation cycle of the powder (2.5 mg) aerosol produced using the PreciseInhale system at an air flow rate of 1.2 L min^−1^. The amount of drug deposited on the coverslips was in the range from 0.99 to 1.20 mg with a mass median aerodynamic diameter (MMAD) of 1.7 mm for BD and of 3.4 mm for FP.

### 3.3. In Vitro Dissolution Methods

In vitro dissolution studies by conventional dissolution methods using USP apparatus 1 (basket) [65,66] and 2 (paddle) [27,67,68,69] have several limitations. Primarily these methods provide well-stirred environments contrasting with the in vivo condition in the alveolar region of the lungs. Homogenous dispersion of the particles into the vessel/basket is challenging, and dispersed particles adhere to the dissolution apparatus components and inadvertently enter the aliquots during the sampling procedure. To make up for some of the deficits of commercial USP 1 and 2 dissolution systems, various in vitro dissolution methods using compendial (USP 2) paddle apparatus, flow-through cell apparatus, dialysis bag, Franz diffusion cell, Transwell^®^ and DissolvIt systems (Table 2) have been developed and applied to evaluate the drug release characteristics of the inhaled dry powder even though they are limited in mimicking the in vivo situation.

#### 3.3.1. Modified USP 2 (Paddle over Disc) Apparatus

**Figure 4:**
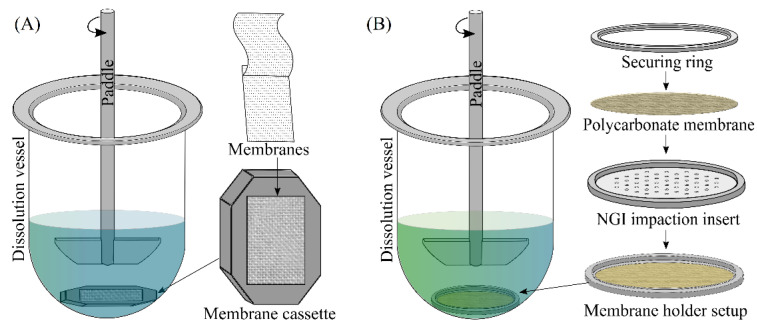
Schematic of paddle-over-disc apparatus with (**A**) membrane cassette, (**B**) NGI membrane holder. (**A**) reproduced with permission from Son and McConville 2009 [28], Elsevier. (**B**) reproduced with permission from Son et al., 2010 [50] Dissolution Technologies, Inc.

The paddle-over-disc dissolution setup consists of a round bottom glass vessel of 150 mL capacity with rotating mini-paddles and a membrane cassette. The membrane cassette is a powder holding device contains two membranes with sandwiched powder particles inside a modified histology cassette frame. Son and McConville 2009 [28], evaluated the dissolution properties of hydrocortisone inhalable powders using a mini-paddle dissolution apparatus containing a membrane cassette (Figure 4). Aerodynamically classified particles collected over a polycarbonate membrane (PC) using NGI were sandwiched using another pre-soaked PC membrane and inserted into the cassette frame. Then, this membrane cassette was placed into a dissolution vessel containing 100 mL of dissolution medium (SLF and mSLF; 37 °C), and drug release was evaluated at a paddle rotation speed of 50 rpm. In this method, the sandwiched dispersed particles in the membrane cassette undergo dissolution in the small volumes of the medium that enters through the pores in the membrane followed by diffusion of the dissolved drug into the bulk reservoir medium. This new method of dissolution studies showed a significant difference in the dissolution profiles between bulk hydrocortisone (HC) and an aerodynamically classified HC. However, the dissolution tests were performed for only a portion of dose collected on the rectangular portion of the membrane due to the holder frame limitations.

Later this research group designed a new, easy to use membrane holder (Figure 4B) to evaluate the dissolution behaviour of the whole dose collected in each NGI plate [50,51]. This new membrane holder consists of a NGI dissolution cup with a removable impaction insert, a securing ring, two sealing o-rings, and a PC membrane. A pre-soaked PC membrane was placed over the impaction insert with dispersed particles and secured in the designed membrane holder. The secured membrane holder with sandwiched particles was transferred into the dissolution vessel containing 300 mL of dissolution medium with membrane side up facing the rotating paddle.

#### 3.3.2. Dialysis Bag

**Figure 5:**
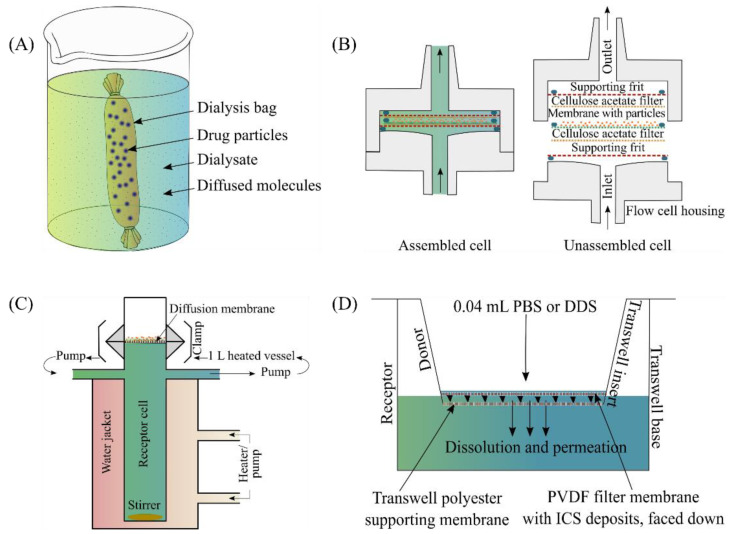
Schematic diagrams of (**A**) dialysis bag method, (**B**) flow-through cell [21], (**C**) Franz diffusion cell [27] and (**D**) Transwell^®^ system [20].. (**B**) reproduced with permission from Davies and Feddah 2003 [21], Elsevier. (**C**) reproduced with permission from Salama et al., 2008 [27], Elsevier. (**D**) reproduced with permission from Arora et al., 2010 [20], Springer Nature.

Dialysis is a separation technique which works by diffusion, a process that results from the thermal motion of molecules in solution from a region of higher to lower concentration until an equilibrium is reached. A dialysis bag made of a semipermeable membrane and had small pores. The bag filled with solid dry powder particles is suspended in a dialysate medium (Figure 5A). The large dry powder particles cannot pass through the pores of the membrane. Upon dissolution of the dry powder particles, the drug molecules are small enough to diffuse through the pores of the membrane into the dialysate medium.

Arora et al., 2015 [72] investigated the voriconazole release from the polylactide microparticles by the dialysis bag (MWCO: 12 kDa) method using 20 mL of phosphate-buffered saline (pH 7.4, 10 mM) containing 0.1% Tween 80 at 37 ± 0.5 °C and 900 rpm. Several other researchers also used dialysis bag method to evaluate the drug release from dry powder particles [73,74,75].

#### 3.3.3. Flow-Through Cell Apparatus

The flow-through dissolution system (Figure 5B) was introduced in 1957 as a flowing medium dissolution apparatus [83] and in 1990, it was officially accepted by the United States, and European Pharmacopoeia for the evaluation of drug release behaviour from various dosage forms. The flow-through cell apparatus is a modified USP apparatus 4 which comprises a filter holder containing a membrane with the loaded powder particles and a pump that forces the dissolution medium from a reservoir into a vertically positioned flow cell. The flow-through system has several advantages: maintenance of sink conditions by the continuous flow of fresh medium; reduced influence of diffusion during dissolution testing [84].

Kanapilly et al., 1973 [85] evaluated the in vitro dissolution patterns of radioactive aerosol particles using two flowing systems (a flow-through system and parallel flow system) with adjustable solvent flow rates and a static system. In the flow-through systems, the aerosol particles were collected on a 0.8 μm membrane filter (sample filter) using a 7-stage round jet cascade impactor and sandwiched between a 0.1 μm membrane (backup filter) and 0.8 μm membrane filter (top cover filter) in a high-pressure steel filter holder. The solvent was pumped vertically to flow through the top cover filter, sample filter, and the backup filter.

Davies and Feddah 2003 [21] adapted the flow through system for in vitro dissolution testing of dry powder particles. In this study, aerodynamically classified aerosol particles were collected over membrane filters using ACI as described earlier and sandwiched using another membrane with a Teflon ring (1 mm thickness) in between the membranes. These sandwiched membranes were placed into the flow through cell held by two stainless steel support filters at both ends and dissolution medium was pumped at a flow rate of 0.7 mL/min in the upward direction to flow through the particles sandwiched in between the membranes.

Taylor et al., 2006 [76] prepared sustained release respirable spray coated particles of ipratropium bromide and evaluated for in vitro drug release behaviour using a flow-through cell method. In this study, the powder samples were placed directly onto a wire mesh screen and inserted into a flow cell of 22.6 mm in diameter. Deionized water (pH 5.5; 37 °C) was passed through the flow cell using a Sotax CY7 piston pump.

#### 3.3.4. Franz Diffusion Cell

Another method which has been commonly used to investigate the in vitro dissolution of inhaled dry powder particles is the Franz diffusion cell. The Franz diffusion cell consists of two compartments, donor and receptor compartments, separated by a membrane as shown in Figure 5C. The donor compartment is exposed to the air while the receptor compartment filled with dissolution medium. The dissolution medium in the receptor compartment is continuously mixed with a magnetic stir bar. An advantage of the Franz diffusion cell is that it provides an air-liquid interface, as present in the lung [25]. However, in a Franz diffusion cell, it may be difficult to distinguish between dissolution rate and diffusion effects through the membrane [86].

A modified Franz diffusion cell was used by Salama et al. [27] to conduct the dissolution test for controlled released microparticles containing disodium cromoglycate (DSCG) and polyvinyl alcohol (PVA) for inhalation. This study compared several different methods of dissolution and found that a modified Franz diffusion cell was able to discriminate dissolution rates better than the flow through cell and the USP apparatus 2.

In another study, May et al. [25] conducted dissolution studies for unnamed drug substance A dibromide and amorphous base, fenoterol and budesonide in PBS (pH 7.4, 1 L) at 37 °C using a modified Franz diffusion cell. A regenerated cellulose membrane filter with a pore size of 0.45 µm was placed into the membrane holder, with the particles collected using the NGI facing upwards. In contrast to the results of Salama et al. [27], this study found that although the Franz diffusion cell was able to discriminate between substances of different solubilities in the dissolution media, it was not as sensitive and as reproducible as the USP dissolution apparatus 2. They speculated that the possible reasons for the variation in the results might be due to the difference in the method set up, membrane type and thickness, and loading dose.

#### 3.3.5. Transwell^®^ System

The Transwell^®^ system (Figure 5D) consists of an upper (donor compartment) and lower (acceptor compartment) chambers separated by a membrane which is made out of either polyester (PET) or polycarbonate (PC) or collagen coated polytetrafluoroethylene (PTFE). Such systems are used in drug transport studies to characterize the permeability in apical-to-basolateral direction. Due to the lower volume of dissolution medium, the Transwell^®^ system could provide more bio-relevant conditions in comparison to the Franz diffusion cell.

Arora et al., 2010 [20] developed a dissolution method for size classified respirable particles using a Transwell**^®^** system with limited volumes of stationary aqueous dissolution medium. Aerodynamically dispersed particles were collected on the filter membrane as reported by Davies and Feddah [21] from stage 4 (2.1–3.3 μm) and stage 2 (4.7–5.8 μm) of compendial Andersen cascade impactor. Then, the filter membranes with deposited aerosol powder were placed with particles facing in the downward direction on a semi-permeable polyester membrane of the Transwell^®^ insert. Immediately after these inserts were transferred into a receptor compartment containing 1.4 mL of dissolution medium, 0.04 mL of the dissolution medium was placed in the donor compartment to initiate the particle dissolution. The system was then placed in an incubator maintained at 37 °C and an aliquot of 0.5 mL was collected (with replacement) from the receptor compartment at different time. At the end of the experiment, the donor compartment was thoroughly washed to recover the undissolved portion of the drug.

Rohrschneider et al., 2015 [26] evaluated the in vitro dissolution behaviour of orally inhalable products using a commercial Transwell^®^ system with polycarbonate membrane and a modified Transwell^®^ system in which polycarbonate membrane was replaced with a glass microfiber filter. In a modified system, incorporating a more permeable membrane, the drug transfer from donor to acceptor compartment was limited by the dissolution of the particles and not by the diffusion through the membrane.

#### 3.3.6. DissolvIt System

**Figure 6:**
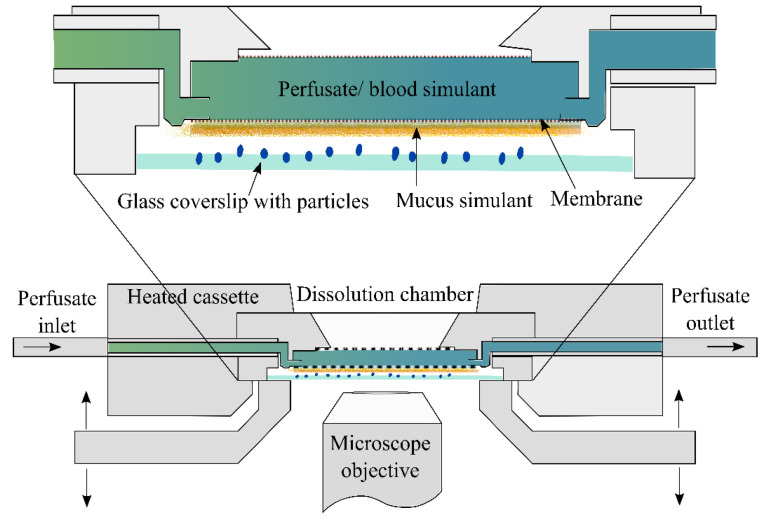
Schematic diagram of DissolvIt^®^ system. Reproduced with permission from Börjel et al., 2014 [59], Respiratory Drug Delivery 2014, Virginia Commonwealth University.

Dissolv*It* system (Figure 6) is a recent in vitro dissolution testing method which simultaneously determines the dissolution and absorption of a drug from respirable size dry powder particles [23]. The system consists of a mucus layer (50 μm thick 1.5% *w/v* poly-ethylene oxide in 0.1 M PBS) on a polycarbonate membrane to mimic the air-blood barrier in the tracheobronchial region of the lung with a blood simulant (0.1 M PBS containing 4% *w/v* albumin) flowing on the other side of the membrane. The fine particle dose (0.99 to 1.20 μg) of respirable size particles was collected on glass coverslips using the PreciseInhale system as discussed in Section 3.2.4. The dissolution behavior of respirable size particles of budesonide and fluticasone propionate was studied by simultaneous observation of particle disappearance under microscope and quantification of drug in the perfusate on the vasular side of the membrane. This new system, in which the blood simulant buffer pumped in singlepass mode through the dissolution cell, enables the generation of in vitro dissolution/absorption curves of drugs from inhaled dry powders.

In a recent study, Dissolv*It* system was used to assess the impact of dissolution medium on dissolution of fluticasone proprionate aerosol particles. A synthetic simulated lung lining fluid, 1.5% poly(ethylene oxide) + 0.4% L-alphaphosphatidyl choline and Survanta were three different media used in the Dissolv*It* chamber. It was illustrated that biorelevant dissolution studies can generate input parameters for physiologically based pharmacokinetic modeling of inhaled drug products [87].

#### 3.3.7. Custom Made Flow Perfusion Cell

Eedara et al. [58] custom made a flow perfusion cell which resembles an air-blood perfusion model to evaluate the dissolution behaviour of respirable size particles. The flow perfusion cell was connected to a syringe pump (100 DM syringe pump, Teledyne ISCO, Lincoln, NE, USA) to collecte the perfusate and an optical microscope equipped with a digital camera (OPTIKA SRL, Ponteranica BG, Italy) to capture the images of respirable size particle dissolution [18,58,62,62]. Using this apparatus, the dissolution behaviours of fine particle doses (collected using mTSI) of moxifloxacin and ethionamide in 25 µL of mucus simulant were evaluated. The respirable size particles of moxifloxacin dissolved quickly (<30 min) compared to the ethionamide.

Similarly, Saha et al. conducted in vitro experiments using custom made flow perfusion cell and showed that ~68% of ivermectin got permeated in 30 h from dry powder formulation. The dissolution medium was polyethylene oxide (1.5% *w*/*w*) + Curosurf^®^ (0.4% *w*/*w*) in phosphate-buffered saline (PBS) and Tween 80 (0.2% *w*/*v*) in PBS was perfusate. 50 μL of the dissolution medium was loaded on the apparatus and the flow rate of the perfusate was fixed at 0.05 mL/min [88].

A major drawback of the dialysis bag, Franz-type diffusion cell, Transwell^®^, Dissolv*It*^®^ and custom made flow perfusion cell methods is that the mass of the drug released into the donor compartment is limited. The advantages and disadvantages/limitations of all the above methods are summarized in Table 3. Even though various dissolution apparatus has been developed for inhaled dry powder particles, maintaining very limited volumes of the dissolution media to simulate the lung conditions is still a challenge. Therefore, the development of a standardized in vitro dissolution method for dry powder particles is still an interesting topic to research.

It has always been fascinating to explore the interactions of inhaled drugs and components of RTLF that ultimately affect their dissolution and absorption in the lungs. For instance, Langmuir monolayer technique enables the formation of a lipid film on the water subphase and facilitates characterization of lipid–water, lipid–lipid or lipid–drug interactions [89]. However, understanding the interactions of inhaled drug molecules and RTLF components is out of the scope of this current review.

## 4. Models for Pulmonary Drug Absorption

In vitro, ex vivo and in vivo models are commonly used to study absorption of inhaled drug particles. Table 4 summarizes the various models used to study pulmonary drug absorption.

In vitro air-to-blood barrier is reconstructed using cell models in the Transwell or Snapwell system under cell culture [96]. Table 5 summarizes different types of cells used for in vitro lung barrier models. Stem cell-derived lung epithelial cells and “lung-on-a-chip” models have grabbed the interest of many researchers. Most importantly, differentiation of human embryonic stem cells (ESC) or induced pluripotent stem cells (iPSC) to alveolar epithelial type II-like cells facilitates large-scale alveolar epithelial cell production. Air-liquid interface (ALI) culture can induce differentiation further to alveolar epithelial type I-like cells. Furthermore, a microfluidic device, “lung-on-a-chip” has been developed as lung model to study biological development and pathogenic responses of lungs. The utility of a unique six-well “lung-on-a-chip” prototype that can integrate an in vitro aerosol deposition system is currently being examined. This attempt looks interesting as it includes the presence of air, media flow and breathing-like stretching that resembles the movement of lungs [96].

When in vivo or in vitro models cannot clearly explain the mechanism of drug transport or lung disposition kinetics, ex vivo tissue models are used. Isolated perfused lung (IPL) is one of the most used method, where the lung is isolated from the body and kept in an artificial system maintaining certain experimental conditions. This separates distribution, metabolism and elimination from lung specific assessments. Architecture and functionality of the tissue is closely maintained in an isolated organ experiment enhancing its resemblance to in vivo state in comparison to in vitro monolayer models from a single cell type. An IPL prepared from small rodents has commonly been employed for lung disposition studies [97].

In vivo studies in intact animal models are used for investigating the absorption, distribution, and pharmacodynamics of inhaled drug particles. In such models, formulations are administered to conscious or anesthetized animals using different types of delivery devices with or without surgical intervention. Small animals such as mice and rats have been commonly used to study pulmonary pharmacokinetics. However, because of higher cost and logistics required for handling and housing, the use of larger animals such as guinea pigs, rabbits, dogs, sheep and monkeys are limited [98]. In larger animals, regional delivery/distribution of drugs can be achieved by the appropriate selection of aerosol size and inspiratory manoeuvres facilitating the study of region dependent lung absorption and disposition [96].

## 5. Future Perspectives

We have summarized various instruments and methods used for dissolution studies of inhaled drug particles, however there is no standard method that can be recommended for routine studies. Therefore, there is a need for sophisticated instrument for testing inhalable formulations. Moreover, currently available small volume dissolution apparatus only accounts for dissolution studies in stagnant medium (simulated RTLF) ignoring the fact that mucociliary clearance occurs in the upper airways and breathing results in the movement of alveoli and air sacs of the lungs. Therefore, small volume dissolution instruments should be developed or upgraded in such a way to incorporate fluid movement in them [30]. In addition, in vitro cell-based models that are being used for absorption studies are inconvenient to conduct routine testing of formulations. Therefore, automated cell free systems are always preferred over them.

On the other hand, various simulated RTLFs (dissolution media) [99] that are being used for in vitro dissolution studies do not closely resemble the human RTLF [30]. Composition and thickness of RTLF vary regionally from one region of respiratory tract to another and individually from healthy to diseased. RTLF is rich in mucus in upper respiratory tract whereas, it is rich in surfactant in lower respiratory tract [30]. For instance, in pulmonary disease like cystic fibrosis (CF), patients have highly tenacious (adhesive and cohesive) sputum. Along with mucin (regular component of normal mucus), CF sputum contains large amounts of DNA and filamentous actin [100] in comparison to RTLF of healthy person. Therefore, there is a need for region-specific and disease-specific simulated RTLFs. Determination of absolute concentration of components of RTLF is a must to mimic them but it is a challenging task. Therefore, there is a need for sophisticated method (technology) to accurately determine them. Moreover, components of simulated RTLF should always be chosen keeping in mind about their cost and availability that in turn will help commercialization in future [30].

## 6. Conclusions

In vitro dissolution testing is a well-established quality control test in characterizing the performance of a solid oral dosage form. However, no approved methods are available for evaluating the dissolution behaviour of inhaled dry powder particles even though many studies proved the relationship between dissolution and pharmacokinetics of inhaled drugs. The complex nature of the lungs with anatomical and physiological differences in the tracheobronchial region and alveolar region make a great challenge in the development of an in vitro dissolution method which mimics the lung conditions.

In this review, we summarized various dissolution methods and absorption models developed for the evaluation of dissolution and absorption behaviour of inhaled drug particles. Even though the recent methods used a small volume of dissolution medium, it represents only a particular region of the lung. A further improvement in the dissolution methods which mimic the different regions of the lungs is necessary.

## Figures and Tables

**Table 1:** Summary of the fine particle dose (FPD) collection methods for dissolution testing of dry powder particles.

Apparatus	Drugs	Inhaler and Loading Dose	Collection Method	Ref.
Andersen Cascade Impactor (ACI)	Budesonide (BD), Fluticasone propionate (FP), Triamcinolone acetonide (TA)	Pulmicort Turbuhaler, BD, 200 µg Flixotide Accuhaler^®^, FP, 250 µg Azmacort^®^, TA, 200 µg	Collected onto a GF filter at the connection point of the induction port and inlet of ACI	[21]
	Flunisolide (FN), TA, BD, FP, Beclomethasone dipropionate (BDP)	Aerobid^®^, FN, 250–2500 µg Azmacort^®^, TA, 200–2000 µg Pulmicort Turbuhaler^®^, BD, 50–500 µg Flovent^®^ HFA and Diskus, FP, 150–1250 µg Vanceril^®^ and QVAR^®^ (BDP, 350–700 µg)	Collected onto 6 PVDF membranes placed at the stage 4 of ACI operated at an air flow of 28.3 L/min	[20]
	BD, Fenoterol HBr (FNH), Substance A dibromide (SAD), Substance A crystalline base (SAC), Substance A amorphous base (SAA)	Micronized BD, FN, SAD and SAC; spray dried SA (SAA) HandiHaler^®^ (1 mg (BD, SA) 10 mg (FN))	Collected onto the RC membrane (pore size 0.45 µm) at standard USP conditions (4 kPa, 4 L)	[25]
	BD, SAD, SAC, SAA	Micronized BD, SAD and SAC; spray dried SA (SAA) HandiHaler^®^ (0.5 to 4 mg)	Collected onto the RC membrane at standard USP conditions (4 kPa, 4 L) using ACI with stage extension between stage 1 and filter stage, and modified/standard filter stage	[47]
	BD, SAD, SAC, SAA	Micronized BD, SAD and SAC; spray dried SA (SAA) HandiHaler^®^ (0.5 to 4 mg)	Collected onto the PE, PC, IPC and RC membranes at standard USP conditions (4 kPa, 4 L) using ACI with stage extension between stage 1 and F, and modified/standard filter stage	[48]
	BD, Ciclesonide (CIC), FP	Symbicort^®^ (BD) Alvesco^®^ (CIC) Flixotide^®^ (FP), (5 doses (BD-80 µg/dose, CIC-60 µg/dose and FP-110 µg/dose))	Collected onto the 24 mm GF filters or Fisherbrand Q8 filter papers at the stage 4 of ACI at an air flow of 28.3 L/min	[26]
	Salbutamol sulfate (SS), FP, Salmeterol xinafoate (SX)	Micronized SS blend, Rotahaler^®^ (6–10 doses (2% w/w SS blend, 30 mg/dose) Seretide^®^ 50/100 Diskus^®^ (FP and SX, 50 µg SX and 100 µg FP/dose))	Collected onto an adhesive tape using the truncated ACI with a PTFE funnel and a collection plate at the filter stage (Stage F) operated at a pressure drop of 4 kPa at 60 L/min air flow rate	[49]
Next Generation Impactor (NGI)	Hydrocortisone (HC)	Bulk HC (50 mg) micronized HC blend Aerolizer^®^ (150 mg micronized HC blend, ~10 mg of HC)	Collected onto the PC (0.05 µm) and CA (MWCO 3500, 12,000) at each dose plate of NGI	[28]
	Albuterol sulfate (AS), BD	Ventolin HFA^®^ (AS,15–20 doses) Pulmicort Flexhaler^®^ (BD, 1–10 doses)	Collected onto the impaction inserts at stage 2–5 of NGI at 30 L/min (AS) or 60 L/min (BD) air flow rate	[50]
	Rifampicin (RIF)	Microparticles; Aerolizer^®^ (7 mg to 20 mg)	Collected onto the impaction insert at stage 3 of NGI operated at 60 L/min air flow rate for 4 s	[51]
	Itraconazole (ITZ)	Spray dried solid dispersions, Axahaler^®^	Collected onto the impaction inserts at each dose plate of NGI operated at 60 L/min air flow rate	[52]
	Tobramycin Clarithromycin	Nanoparticulate spray dried powders (TCn2), Physical blend (TCb), Axahaler^®^	Collected onto the impaction insert at stage 3 of NGI operated at 100 L/min air flow rate	[53]
	Pyrazinamide (PYR), RIF, Isoniazid (IZD)	Spray dried powders, Aerolizer^®^ (Two 20 mg doses)	Collected onto a NC membrane at stage 3 of NGI operated at 100 L/min air flow rate	[54]
	FP	Flixotide^®^ (FP, 5 doses of 110 µg/dose)	Collected onto the 24 mm Fisherbrand Q8 filter papers at stage 2 and 4 of NGI	[26]
Twin Stage Impinger (TSI)	Dextrans labelled with fluorescein isothiocyanate (FITC-dex; 4, 10, 20, 40 and 70 kDa)	A custom-made glass dry powder insufflator (5 mg)	Collected onto the Calu-3 bronchial epithelial cells in a Transwell^®^ insert using TSI at 60 L/min air flow rate for 5 s	[55]
	BDP	QVAR^®^ and Sanasthmax^®^ (100–250 μg/dose; 1.2 ± 0.12 mg deposited)	Collected on a NC membrane (0.45 μm) at stage 2 of a modified TSI	[56]
	Salbutamol base (SB), SS	Micronized SB and SS (5 mg)	Collected onto a Transwell^®^ PE insert (0.4 μm) using modified TSI at 60 L/min air flow rate for 4 s	[57]
	Moxifloxacin Ethionamide	Aerolizer^®^ device (20 mg)	Collected on a glass coverslip at 60 L/min air flow rate for 4 s	[58]
PreciseInhale system	BD, FP	Micronized powders (2.5 mg)	Collected onto the glass coverslips of 13 mm diameter at 1.2 L/min air flow rate	[23,59]

CA—cellulose acetate; GF—glass fibre; IPC—isopore polycarbonate; NC—nitrocellulose; PC—polycarbonate; PE—polyester; PTFE—polytetra fluoroethylene; PVDF—polyvinylidene difluoride; RC—regenerated cellulose; USP—united states pharmacopoeia.

**Table 2:** Summary of various in vitro dissolution testing methods for dry powder particles.

Dissolution Apparatus	Membrane (Pore Size, μm or MWCO, kDa)	Dissolution Medium and Conditions	Ref.
USP 1 (basket) apparatus	Glass fiber filters, GF/F grade	PBS, pH 7.4, basket rotation- 150 rpm	[65]
	-	PBS, pH 7.4, 900 mL, basket rotation- 100 rpm	[66]
USP 2 [paddle) apparatus	-	Water, 300 mL, paddle rotation- 50 rpm	[68]
	-	Buffer, pH 1.2 or pH 6.8, 1000 mL, paddle rotation- 100 rpm	[67]
	-	PBS, pH 6.8, 1000 mL, paddle rotation- 50 rpm	[69]
Modified USP 2 (paddle over disc) apparatus	-	PBS, pH 7.4, 1000 mL, paddle rotation- 50 rpm	[27]
	Polycarbonate membranes (0.05 and 1 μm) Cellulose acetate membranes (3.5, 12 kDa)	SLF and modified SLF with DPPC (0.02% *w*/*v*), pH 7.4, 100 mL, paddle rotation- 50 rpm	[28]
	Polycarbonate membrane (0.05 μm)	SLF, 0.2 M PB, pH 7.4, PBS, modified PBS with DPPC, tween 80 (0.02 and 0.2% *w*/*v*), pH 7.4, 300 mL, paddle rotation- 50, 75, 100 rpm	[50]
	Polycarbonate membrane (0.05 μm)	PBS, pH 7.4 or 0.2 M citrate buffer with ascorbic acid (0.02% *w*/*v*), pH 5.2, 300 mL, paddle rotation- 75 rpm	[51]
	Polycarbonate membrane (0.4 μm)	Water with SLS (0.3%), buffer, pH 1.2, 300 mL, paddle rotation- 75 rpm	[52]
	Regenerated cellulose membrane (0.45 μm)	PBS, pH 7.4, 1000 mL, paddle rotation- 50, 100, and 140 rpm	[25,47]
	Polycarbonate membrane (0.4 μm)	PBS, pH 7.4, 300 mL, paddle rotation- 75 rpm	[53,70]
	Dialysis membrane (>900 kDa)	Gamble’s solution, pH 7.4 and alveolar lung fluid, pH 4.5, 900 mL, paddle rotation- 150 rpm	[71]
	-	Modified SLF with tween 80 (0.2% *v*/*v*), 50 mL; paddle rotation- 50 rpm	[49]
Dialysis bag	Dialysis membrane (12 kDa)	10 mM PBS with tween 80 (0.1% *v*/*v*), pH 7.4, 20 mL, rotation- 900 rpm	[72]
	Dialysis membrane (12–14 kDa)	SLF, pH 7.4, 50 mL	[73]
	Dialysis membrane (12–14 kDa)	SLF, pH 7.4, 30 mL,	[74]
	Dialysis membrane (14 kDa)	PBS, pH 7.4, 250 mL, rotation- 100 rpm	[75]
Flow-through cell system	Cellulose acetate membrane (0.45 μm)	SLF, modified SLF with DPPC (0.02% *w*/*v*), flow rate- 0.7 mL/min,	[21]
	-	Deionized water, pH 5.5, medium flow rate- 5–16 mL/min,	[76]
	Nitrocellulose membrane (0.45 μm)	0.05 M PBS, pH 7.4, 1000 mL, medium flow rate- 0.5 mL/min	[27]
	Regenerated cellulose membrane (0.45 μm)	PBS, pH 7.4, medium flow rate- 0.5 mL/min	[25]
Franz diffusion cell	Nylon membrane (0.45 μm)	Degassed 0.05 M PB, pH 7.4, 17.5 mL, rotation- 240 rpm	[77]
	Nitrocellulose membrane (0.45 μm)	0.05 M PBS, pH 7.4, 1000 mL, medium flow rate- 5 mL/min	[27]
	MF™ membrane (0.45 μm)	PB, pH 7.4, 250 mL, medium flow rate- 5 mL/min	[78]
	Nitrocellulose membrane (0.45 μm)	PB, pH 7.4 containing 0.1% *w/v* SDS	[56]
	Polyester membrane (0.4 μm)	HBSS or SLF with DPPC (0.02% *w*/*v*), 50 mL, medium flow rate- 5 mL/min	[57]
	Regenerated cellulose membrane (0.45 μm)	PBS, pH 7.4, 1000 mL, magnet rotation- 100 rpm	[25]
	Regenerated cellulose membrane (0.45 μm)	Water, PB, pH 7.4, or modified SLF, pH 7.4, 10 mL, 75 rpm	[79]
	Cellulose acetate membrane (0.2 μm)	0.05 M degassed PB, pH 7.4, or SLF, 0.15 mL in donor compartment and 27 mL in receiver compartment	[80]
	Nitrocellulose membrane (0.45 μm)	SLF, pH 7.4, 22.7 mL	[54]
	Polycarbonate membrane (0.4 μm)	SLF with SDS (0.5% *w*/*v*), 4.2 mL	[81]
	Filter paper	PBS, pH 7.4, 21.5 mL	[82]
Transwell^®^ system	Polyester membrane (0.4 μm)	PBS, pH 7.4 or distilled deionized water, pH 7.0, 0.04 mL in donor compartment and 1.4 mL in well plate	[20]
	Polycarbonate membrane (0.4 μm) or Polyester membrane (0.4 μm)	PBS, pH 7.4, 2.6 mL or 3.85 mL	[48]
	Polyester membrane (0.4 μm)	PBS with SDS (0.5% *w*/*v*), 0.1 mL in donor compartment and 1.5 mL in well plate	[26]
Dissolvit^®^	Polycarbonate membranes (0.03 μm)	1.5% *w/v* PEO in 0.1 M PB with DPPC (0.02 and 0.4% *w*/*w*)	[23]
Custom-made flow perfusion cell	dialysis membrane (MWCO = 12,400 Da)	1.0, 1.5, 2.0% *w/v* PEO in PBS, pH 7.4 1.5% *w/v* PEO in PBS, pH 7.4 with Curosurf^®^	[58]

DPPC—di-palmitoylphosphatidylcholine; HBSS—Hanks balanced salt solution; PB—phosphate buffer; PBS—phosphate-buffered saline; PEO—polyethylene oxide; SDS—sodium dodecyl sulfate; SLF—simulated lung fluid; SLS—sodium lauryl sulfate.

**Table 3:** Advantages and disadvantages/limitations of the apparatus used to evaluate the dissolution behaviour of the inhaled products. Reproduced with permissions from Eedara et al., 2019 [58], Elsevier.

Apparatus	Advantages	Disadvantages/limitations
Paddle over disc apparatus	- Easy handling - Sink conditions maintained by large dissolution medium volumes	- Absence of an air-liquid interface - Large volumes of the dissolution medium and paddle agitation are not reflective of the actual in vivo dissolution process of inhaled particles
Dialysis bag	- Static and sink conditions maintained - Dissolution medium replacement possible	- Absence of an air-liquid interface - The powder particles might adhere to the sides of the bag or aggregate
Flow through cell	- Sink conditions maintained by the continuous flow of fresh medium - Reduced influence of diffusion during dissolution testing - Continuous sampling, a flow rate change and dissolution medium change possible during the run	- Absence of an air-liquid interface - The high fluid velocity applied does not represent the agility of the fluid in the lung, which is rather stationary - The flat geometry of the filter holders potentially generates a high fluid velocity at the centre but decreasing flow gradient towards the periphery causing diffusion effects and a local non-sink condition - Handling of this setup is sensitive to entrapped air in the membrane-drug substance sandwich and in the dead volume of the flow-through cell - The drug release is flow-rate controlled
Franz diffusion cell	- Represent the in vivo non-agitated situation - Consists of an air-liquid interface	- Presence of air bubbles at the membrane liquid interface and the difficulty or even failure of removing them - Difficult to distinguish between diffusion effects through the membrane and the dissolution rate
Transwell^®^ system	- Represent the in vivo non-agitated situation - Consists of an air-liquid interface - Lower volumes of stationary dissolution media	- Difficult to distinguish between diffusion effects through the membrane and the dissolution rate - Saturation of the limited volume of dissolution medium causes decreased dissolution
Dissolv*It*^®^ system	- Simulates the air-blood barrier of the upper airways of the lungs with low volumes of stationary mucus simulant - Particle dissolution is visualized under microscope as disappearance - Simulates the dissolution and absorption of drugs from inhaled dry powders	- The microscopic observation is limited by an optical resolution of around 0.2 μm - The thickness of the stationary mucus and membrane simulates the absorption kinetics in the tracheobronchial region rather than the deep lung regions

**Table 4:** Summary of various models for pulmonary drug absorption.

Models		Drugs	Ref.
In vitro	Air-liquid interfaced layers Calu-3/Transwell system	Salbutamol Indomethacin	[90]
	DissolvIt system	Budesonide Fluticasone propionate	[23]
	Custom made flow perfusion cell	Moxifloxacin Ethionamide	[58]
Ex vivo	Isolated perfused rat lung	AZD5423 (developmental nonsteroidal glucocorticoid) Budesonide Fluticasone furoate Fluticasone propionate	[91]
In vivo	Rats	Rifampicin	[92]
	Guinea pigs	Rifampicin	[93]
	Cynomolgus monkeys (non-human primates)	Erythropoietin Fc fusion protein	[94]
	Patients with cystic fibrosis (Clinical study)	Colistin	[95]

**Table 5:** In vitro models for absorption of inhaled drug particles [33].

Alveolar Epithelial Models	Tracheobronchial Epithelial Models
Primary alveolar epithelial cell cultures	Primary cell cultures Small airway epithelial cells Normal human bronchial epithelial cells
Alveolar epithelial cell lines A549 NCI-H441 human bronchiolar epithelial cell	Bronchial epithelial cell lines BEAS-2B NuLi-1 16HBE14o Calu-3 Models of cystic fibrosis airway epithelium (NCF3, CFT1, CFBE41o, CuFi)
	Co-culture models or human bronchial/alveolar cells
